# Treating Metastatic Brain Cancers With Stem Cells

**DOI:** 10.3389/fnmol.2021.749716

**Published:** 2021-11-24

**Authors:** Nadia Sadanandan, Alex Shear, Beverly Brooks, Madeline Saft, Dorothy Anne Galang Cabantan, Chase Kingsbury, Henry Zhang, Stefan Anthony, Zhen-Jie Wang, Felipe Esparza Salazar, Alma R. Lezama Toledo, Germán Rivera Monroy, Joaquin Vega Gonzales-Portillo, Alexa Moscatello, Jea-Young Lee, Cesario V. Borlongan

**Affiliations:** ^1^Georgetown University, Washington, DC, United States; ^2^University of Florida, Gainesville, FL, United States; ^3^Department of Neurosurgery and Brain Repair, University of South Florida Morsani College of Medicine, Tampa, FL, United States; ^4^University of Michigan, Ann Arbor, MI, United States; ^5^Michigan State University College of Osteopathic Medicine, East Lansing, MI, United States; ^6^Lake Erie College of Osteopathic Medicine, Bradenton, FL, United States; ^7^Centro de Investigación en Ciencias de la Salud (CICSA), Facultad de Ciencias de la Salud (FCS), Universidad Anáhuac México Campus Norte, Huixquilucan, Mexico; ^8^Universidad Peruana de Ciencias Aplicadas, Lima, Peru; ^9^Center of Excellence for Aging and Brain Repair, University of South Florida Morsani College of Medicine, Tampa, FL, United States

**Keywords:** blood brain barrier, melanoma, brain metastases, stem cell therapy, bone marrow derived mesenchymal stem cell, endothelial progenitor cell, neuroinflammation

## Abstract

Stem cell therapy may present an effective treatment for metastatic brain cancer and glioblastoma. Here we posit the critical role of a leaky blood-brain barrier (BBB) as a key element for the development of brain metastases, specifically melanoma. By reviewing the immunological and inflammatory responses associated with BBB damage secondary to tumoral activity, we identify the involvement of this pathological process in the growth and formation of metastatic brain cancers. Likewise, we evaluate the hypothesis of regenerating impaired endothelial cells of the BBB and alleviating the damaged neurovascular unit to attenuate brain metastasis, using the endothelial progenitor cell (EPC) phenotype of bone marrow-derived mesenchymal stem cells. Specifically, there is a need to evaluate the efficacy for stem cell therapy to repair disruptions in the BBB and reduce inflammation in the brain, thereby causing attenuation of metastatic brain cancers. To establish the viability of stem cell therapy for the prevention and treatment of metastatic brain tumors, it is crucial to demonstrate BBB repair through augmentation of vasculogenesis and angiogenesis. BBB disruption is strongly linked to metastatic melanoma, worsens neuroinflammation during metastasis, and negatively influences the prognosis of metastatic brain cancer. Using stem cell therapy to interrupt inflammation secondary to this leaky BBB represents a paradigm-shifting approach for brain cancer treatment. In this review article, we critically assess the advantages and disadvantages of using stem cell therapy for brain metastases and glioblastoma.

## Introduction

Limited evidence exists on treatments aimed at repairing the blood-brain barrier (BBB) in metastatic brain cancers. According to 2015 data, worldwide cases of melanoma were 351, 880 with Australasia, North America, Eastern Europe, Central Europe, and Western Europe having the highest incidences (Karimkhani et al., 2017). In the U.S, metastatic cancers account for 98,000–170,000 of mass lesions in the brain, which amounts to 24–45% of all cancer patients and 20% of cancer deaths each year (Eisen et al., 2011; Patel et al., 2011; Schwartzentruber et al., 2011; Tu et al., 2011; Zhang et al., 2011; Kaneko et al., 2015; Kotecha et al., 2018; Valiente et al., 2018; Boire et al., 2020). Melanoma is a cancer of the skin with significant morbidity and mortality due to how it readily metastasizes to other areas of the body (e.g., brain) (Kaneko et al., 2015; Schadendorf et al., 2018; Simard et al., 2018; Turner et al., 2018; Gowda et al., 2020). Exploring mechanisms mediating melanoma metastasis advances the basic science understanding of tumor formation and opens avenues for developing novel treatments, including stem cell therapy for brain cancer (Rambow et al., 2018; Wickremesekera et al., 2019; Krishna et al., 2020; Kudchadkar et al., 2020; Zhu et al., 2020). Treating melanoma represents a significant unmet clinical need, as half a million Americans are diagnosed with melanoma each year. Novel strategies, such as stem cell therapy, for treating this melanoma, therefore, deserves serious investigation. Temporary injury to the BBB can allow systemic diseases to seize the opportunity to invade the central nervous system (CNS) (Gerstner and Fine, 2007; Fidler et al., 2010; Fidler, 2011; Sweeney et al., 2019; Profaci et al., 2020) leaving the brain susceptible to further tumor growth (Aragon-Ching and Zujewski, 2007; Palmieri et al., 2007; Kaneko et al., 2015; Osswald et al., 2016; Carmen-Orozco et al., 2019; Schulz et al., 2019; Yang and Torbey, 2020; Huang et al., 2021). An altered permeability in the leaky BBB allows pro-inflammatory molecules to enter the CNS, which may mediate the amelioration of the BBB can potentially decrease pro-tumorigenic effects (Toyoda et al., 2013; Varatharaj and Galea, 2017; Sarkaria et al., 2018; Kwon et al., 2019; Arvanitis et al., 2020).

Invasion of melanoma into the brain correlates with BBB impairment, which leads to the infiltration of inflammatory factors from systemic circulation and a subsequent increase in the malignancy of such cancers. Identifying that this neuroinflammation worsens malignancy, we hypothesize that through angiogenesis, stem cell therapy will repair the leaky BBB, suppress inflammation, and ultimately decrease tumor growth and malignancy in the brain (Kaneko et al., 2015). Our proposal is that stem cell transplantation will hinder brain metastasis of melanoma through repair of the BBB and inhibition of brain inflammation. Of note, there are concerns regarding the use of stem cells and promoting angiogenesis or vasculogenesis in any form of cancer (Ramjiawan et al., 2017; Li S. et al., 2019; Atiya et al., 2020; Unterleuthner et al., 2020). Potentially, stem cells can support tumor growth, provide support for tumors, or become cancerous themselves (Ayob and Ramasamy, 2018; Chae and Kim, 2018; Volarevic et al., 2018; Vakhshiteh et al., 2019; Cable et al., 2020). The cell proliferative process of angiogenesis or vasculogenesis is traditionally viewed as an exacerbating factor for the formation of metastatic cancers; however, our proposition represents a paradigm shift in this perception (Kaneko et al., 2015; Batlle and Clevers, 2017; López-Lázaro, 2018; Schito, 2019; Yi et al., 2019; Dzobo et al., 2020; Lv et al., 2020). As BBB breakdown is exacerbated by decreased angiogenesis and vasculogenesis, promotion of blood vessel growth through bone marrow-derived endothelial progenitor cells (EPCs) may rescue the BBB’s function and attenuate developing brain metastases of melanoma. This hypothesis represents a significant advance over current data on the therapy and pathology of brain cancers (Kaneko et al., 2015; Yang J. et al., 2020).

Angiogenesis, vasculogenesis, and BBB genesis are all distinct phenomena, but are physiologically linked. In cerebrovascular development, blood vessel generation is directed by vasculogenesis and angiogenesis, which are discrete mechanisms (Lee et al., 2009; Yang J. et al., 2020). During development, vasculogenesis generates the primary vascular plexus, which is further developed into a highly intricate vascular network by angiogenesis (Yang J. et al., 2020). Vasculogenesis functions to generate new blood vessels but does so by inducing differentiation of endothelial cell precursors (angioblasts) into mature endothelial cells (Liman and Endres, 2012). Vascular damage triggers vasculogenesis in which bone marrow derived EPCs are stimulated and migrate to the injured site where they mature into endothelial cells (Liman and Endres, 2012). Moreover, damage to the BBB would provoke vasculogenesis with the aim to repair injured endothelial cells of the BBB, thereby mitigating BBB disruption.

Angiogenesis is the process of new capillary formation from existing blood vessels (Rust, 2020). Angiogenesis assists in post-stroke recovery, as the formation of new vessels is crucial for neurogenesis and synaptogenesis (Yang et al., 2018, 2013; 2018). After an ischemic stroke, angiogenesis is initiated in the peri-infarct areas, which has been associated with improved stroke rehabilitation in preclinical models (Nih et al., 2018; Rust et al., 2019; Rust, 2020). Specifically, hypoxia induces endothelial cell damage, triggering the secretion of angiogenic factors, which promotes endothelial cell proliferation for the creation of new blood vessels (Schreiber et al., 2013; Yang et al., 2018, Yang J. et al., 2020). In response to inflammation, mesenchymal stromal cells (MSCs) promote angiogenic and trophic factors that repair and remodel the BBB (Zhang et al., 2020). Pericytes and endothelial cells associated with the BBB influence angiogenesis via secretion of angiogenic and trophic factors (Zhang et al., 2020). With the protection of pericytes, the endothelial cells can promote the generation of pericytes, which in turn can promote the stimulation of newly formed blood vessels and repair of endothelial tight junctions (Zhang et al., 2020). Increased permeability in the presence of inflammation is also influenced by astrocytes withdrawing endfeet interaction with vessels (Park et al., 2015). MSCs may have the ability to increase filaments in the astrocytic endfeet, which may restore interaction with blood vessels and cause the endfeet to extend back (Park et al., 2015). Astrocyte uptake of proinflammatory cytokine may be decreased in the presence of MSCs, thus strengthening the endothelium tight junctions via VEGF-A signaling (Park et al., 2015). These factors together have the potential to repair the leaky BBB while decreasing inflammation. Furthermore, both angiogenesis and vasculogenesis lead to formation of new blood vessels in response to vascular injury but proceed via different mechanisms.

Like vasculogenesis and angiogenesis, barriergenesis is an important component of cerebrovascular development. After angiogenesis completes the formation of the primary vascular network, barriergenesis occurs where cerebral vessels mature into the BBB. This process is specifically mediated by mural cells and the formation of the extracellular matrix (ECM) (Lee et al., 2009; Al Ahmad et al., 2011; Yang J. et al., 2020). As the cerebrovasculature develops, the vessels interact with neurons and glial cells to compose the neurovascular unit (NVU). The crosstalk among endothelial cells, basement membrane, mural cells (e.g., pericytes), neurons, microglia, astrocytes, and oligodendrocytes, all of which function as the NVU, mediate angiogenesis and contribute to barriergenesis (Huang et al., 2020). These interactions among the components of the NVU indicate the link between angiogenesis and barriergenesis. There are signaling pathways, such as Wnt/beta-catenin, in the CNS that induce angiogenesis and also maintain the BBB, specifically upregulating the expression of tight junctions, transporters, and reinforcing BBB permeability (Blanchette and Daneman, 2015; Nguyen et al., 2021). Notably, Wnt/beta-catenin signaling seems to link angiogenesis and BBB maintenance, as the inhibition of this pathway spurs the dysregulation of angiogenesis and BBB impairment (Liebner et al., 2008; Blanchette and Daneman, 2015). In addition, TNF receptors, DR6 and TROY, are expressed in endothelial cells of the brain due Wnt stimulation and mediate barriergenesis and angiogenesis (Tam et al., 2012). Altogether, angiogenesis and vasculogenesis stand as similar mechanisms in the production of new blood vessels, both of which are important for cerebrovascular development and also repair of vascular injury, including BBB damage.

Using angiogenesis and vasculogenesis to repair BBB dysfunction and suppress metastatic brain disease is a controversial hypothesis. As with every other cell type in the body, cancer cells require vasculature for oxygen, nutrients, and removal of waste (Kaneko et al., 2015; Schaaf et al., 2018; Ma et al., 2019; Ballabio and Bonifacino, 2020; Ngoi et al., 2020). As tumors expand, their metabolic demand increases as well, leading to the production of pro-angiogenic factors (Folkman, 1990; Díaz-Flores et al., 1994; Zhang et al., 2012; Becerra and Notario, 2013; Rahma and Hodi, 2019; Ribatti and Tamma, 2019; Teleanu et al., 2019; Vaupel et al., 2019; Tasdogan et al., 2020; Testa et al., 2020). The vasculature associated with a tumor is poorly structured and functionally incompetent due to the disordered and rapid growth triggered by tumor cells, demonstrating an important caveat (Korfel et al., 2002; Graham and Unger, 2018; Petrillo et al., 2018; Zanotelli and Reinhart-King, 2018; Lugano et al., 2020). Often, these hastily constructed vessels are unable to support dependent tissue and lead to microenvironmental hypoxia and ischemia (Jain, 2005; Heath and Bicknell, 2009; Finger and Giaccia, 2010; von Loga and Gerlinger, 2017; Wang et al., 2018; Wang C. et al., 2020; Hinshaw and Shevde, 2019; Boedtkjer and Pedersen, 2020; Lugano et al., 2020). Continuing to inhibit angiogenesis and vasculogenesis exacerbates the damaging microenvironment, preventing repair of the damaged BBB, and ultimately encouraging the pathological growth and formation of melanoma and other metastatic brain cancers (Finger and Giaccia, 2010; Luo et al., 2014; Da Ros et al., 2018; Liebner et al., 2018; Almeida et al., 2019; Gutzmer et al., 2020; Simiczyjew et al., 2020). Repairing the BBB by inducing the processes of angiogenesis and vasculogenesis holds promise as an effective intervention for attenuating brain metastatic cancer development.

## Inflammation-Mediated Breach of Blood-Brain Barrier in Brain Cancer

The BBB is a structure composed of capillary endothelial cells sealed with tight junctions and supported by the neurovascular unit, which consists of not only the endothelial cells but also pericytes, astrocytes, the extracellular matrix, neurons, and microglia (Persidsky et al., 2006; Daneman and Prat, 2015; Eldahshan et al., 2019; Langen et al., 2019). The BBB exhibits selective permeability due to tight junctions between endothelial cells and limited transcytosis due to the expression of selective transporters allowing for the regulation of ion and nutrient passage into the brain (Dejana, 2004; Abbott et al., 2006; Ayloo and Gu, 2019; Segarra et al., 2021). The structural features of the BBB correlate with its function to shield the brain from toxic substances and pathogens and preserve a state of homeostasis in the brain (Langen et al., 2019). The BBB also plays a role in mediating inflammation (Varatharaj and Galea, 2017) and nutrient supply (Dejana, 2004; Abbott et al., 2006; Segarra et al., 2021). The presence of brain tumors impairs BBB functionality and enhances permeability, especially at the location of the tumor (Groothuis et al., 1991; Deeken and Loscher, 2007; Molotkov et al., 2021). Metastatic brain tumors impair BBB functionality by breaking down tight junctions and inducing damage to the NVU, specifically astrocytes and pericytes.

Metastatic tumor cells may infiltrate the BBB in order to overwhelm the brain’s functional tissue. To accomplish this, primary and metastatic brain tumor cells disrupt the structure and utility of the BBB by downregulating tight junction protein expression in endothelial cells, which leads to leakage (Liebner et al., 2000; Papadopoulos et al., 2001; Tran et al., 2019). Tumor cells may also upregulate the expression of proteases, such as cathepsin S, which function to break down adhesion proteins in the BBB (Sevenich et al., 2014; Schulz et al., 2019). In addition, tumor cells diminish BBB functionality by impairing astrocytes and pericytes, which act as key regulators of BBB integrity (Daneman et al., 2010; Arvanitis et al., 2020). Astrocytes function by formulating plasmin upon leakage to combat metastatic invasion (Priego et al., 2018). An *in vivo* administration of the astrocyte toxin 3-chloropropanediol demonstrates how the repopulation of astrocytes in a damaged brain tissue can significantly reduce the fluorescent 10-kDa dextran permeability utilized to portray leakage (Willis et al., 2004; Winkler et al., 2021). Moreover, pericytes regulate the BBB via endothelial cell gene expression, contractile fibers restricting capillary blood flow and transendothelial vesicular transport (Herndon et al., 2017). For example, a lack of pericytes in the endothelium downregulates CD71 (transferrin receptor) expression (Armulik et al., 2010; Herndon et al., 2017). Pericytes can also influence endothelial vesicular trafficking by suppressing Plvap which can affect transcytosis since Plvap is a gene highly expressed during BBB breakdown (Daneman et al., 2010; Wei et al., 2021). Consequently, leukocyte infiltration, edema, and neuronal degeneration result following astrocyte loss, and damaged pericytes compromise astrocytic endfeet polarization and vesicle trafficking (Bush et al., 1999; Armulik et al., 2010; Daneman et al., 2010). Glioma growth and invasion displace pericytes and astrocytic endfeet, damage endothelial cell tight junctions, and alter cell signaling (Dubois et al., 2014; Watkins et al., 2014; Arvanitis et al., 2020). Metastatic brain cells communicate with astrocytes via gap junctions, leading to astrocytic cytokine release that supports tumor progression, chemoresistance, and further BBB breakdown (Chen Q. et al., 2016; Liebner et al., 2018). Through interleukin 1-Beta-mediated NF-kB signaling, these metastatic cells can drive the transformation of healthy astrocytes into tumor associated astrocytes (TAAs) that foster tumor expansion (Xing et al., 2016). Once metastatic cancer cells overwhelm the BBB, the structure develops into a highly variable blood-tumor barrier (BTB), which exhibits various levels of permeability (Babak et al., 2020).

When BBB functionality declines, tumor cells can more easily infiltrate the brain due to decreased selective permeability. High levels of tumor growth create hypoxic areas in the brain due to increased rates of oxygen consumption by tumor cells (Brown and Giaccia, 1998). The hypoxic tumor environment then plays a role in diminishing antitumor immune activity, specifically by suppressing T-cell and Natural killer cell responses (Hatfield et al., 2015; Rankin et al., 2016). On the other hand, hypoxia also generates a heightened inflammatory response that can enhance tumor growth (Finger and Giaccia, 2010; Guo et al., 2016; Salmaninejad et al., 2019). The hypoxic microenvironment from rapid and unregulated tumor growth then recruits macrophages (Finger and Giaccia, 2010; Guo et al., 2016) that release multiple angiogenic factors, including VEGF, which aid tumor extravasation and vascular permeability (Salmaninejad et al., 2019). VEGF and TGFβ1/Smad may interact together to increase angiogenesis in tumor growth, promoted by MSCs (Li et al., 2016). Interestingly, up to 50% of the tumor cells in a glioblastoma mass consist of tumor-associated macrophages, which encompass peripheral macrophages and microglia (Chen et al., 2017). In mice models of glioblastoma, blood levels of monocytes and neutrophils decreased substantially as the tumor progressed, indicating escalated infiltration of these immune cells across the BBB and into the tumor (Chen et al., 2017). Furthermore, macrophage migration to the tumor site seems to exacerbate brain metastases by fostering tumor growth rather than inhibiting it.

Additionally, brain metastatic cells secrete inflammatory cytokines, which recruit peripheral immune cells (e.g., macrophages, neutrophils, lymphocytes), leading to further tumorigenesis and metastasis (Finger and Giaccia, 2010; Xing et al., 2016). Notably, pro-inflammatory cytokines alone can significantly increase BBB permeability (Xiong et al., 2015; Molotkov et al., 2021), which would allow for further tumor cell infiltration into the brain and heightened neuroinflammation. In addition to promoting the proliferation of cells, inflammatory cytokines alter tumor-suppressor gene and oncogene expression, resulting in the attenuation of apoptotic mechanisms (Schetter et al., 2010; Saito et al., 2011; D’Orazi et al., 2021). Specifically, interleukin-6 (IL-6) has been associated with tumor growth and angiogenesis (Sansone et al., 2007; Iliopoulos et al., 2009; Korkaya et al., 2012; Chiou et al., 2013; Li S. et al., 2019). IL-6 has been shown to spur glioblastoma progression via the suppression of miR142-3p expression (Chiou et al., 2013). In patients with brain metastatic lung carcinoma, programmed death-ligand 1 (PD-L1) expressing myeloid cells were significantly higher in count compared to the healthy patients, and increased PD-L1 cell levels were correlated with worse patient outcome (Li Y. D. et al., 2019). Notably, anti-IL-6 antibodies diminished PD-L1 expression, indicating that IL-6 plays a role in the PD-L1- induced immunosuppression associated with tumor expansion (Li et al., 2015). In addition, tumor necrosis factor alpha (TNF-a) has been shown to promote glioma progression via the TNIP1-mediated TNF-a/NF-kB signaling pathway (Lei et al., 2020). Altogether, inflammatory cytokines released by brain metastatic cells and recruited macrophages bolster tumor cell proliferation, vascularization, and BBB leakiness, all of which led to further tumor expansion.

Given the role of the BBB in tumor growth and brain metastasis, restoration of the damaged BBB may serve as a promising therapeutic strategy against metastatic cancer. Amelioration of transcellular BBB leakiness could inhibit the infiltration of additional brain metastatic cells and invading peripheral immune cells, such as inflammatory macrophages. As a result, further tumor expansion and neuroinflammation could be mitigated by chemotherapy with increased BBB permeability presenting as a conducing brain microenvironment, as therapeutic molecules would have less difficulty entering the brain. However, multiple studies indicate that there is no correlation between tumor-induced BBB leakiness and increased efficacy of chemotherapeutic molecules (Rosner et al., 1986; Lee et al., 1989; Fujita et al., 2000; Bernardo et al., 2002; Chen et al., 2002; Korfel et al., 2002; Nisticò et al., 2012; Iwadate, 2016; Ashrafizadeh et al., 2020; Lin et al., 2020). Importantly, the efficacy of therapeutic delivery to the cancer-afflicted brain can be improved upon, potentially through nanoparticles (Ni et al., 2021), high-intensity focused ultrasound (HIFU) (Molotkov et al., 2021), or exosomes (Dong, 2018).

## Stem Cell Rescue of the Pathologic Blood-Brain Barrier

Transplanted bone marrow-derived EPC, specifically modified to target angiogenic and vasculogenic pathways, may prove to be efficacious for BBB repair. Bone marrow-derived stem cells expressing EPC phenotypes, have previously been shown to ameliorate BBB damage and exhibit typical permeability while simultaneously preserving mitochondria and modulating pinocytosis in stroke, as also seen in amyotrophic lateral sclerosis (Borlongan et al., 2004; Garbuzova-Davis et al., 2017, 2018, 2019a, b, 2020; Eve et al., 2018; Saft et al., 2020). Similarly, this method could also be used to restore a leaky BBB observed in metastatic cancers.

Brain metastases can cause BBB breakdown allowing systemic circulating inflammatory mediators to bypass the BBB resulting in increased metastatic brain cancer proliferation (Sprowls et al., 2019; Arvanitis et al., 2020; Garbuzova-Davis and Borlongan, 2021). To illustrate this mechanism, experimental modeling of brain metastatic cancer, such as melanoma, has been executed by infusing into a common inbred strain mouse the mouse melanoma cell line B16F10 via intracarotid injection. Additionally, assessing brain tumor growth, BBB leakiness, and inflammatory response has been achieved through endpoint assays such as *in situ* hybridization, quantitative real-time polymerase chain reaction, and immunoreactive analyses. Considering that this leaky BBB-mediated inflammation worsens metastatic brain cancer outcomes (Bhowmik et al., 2015; Babak et al., 2020; Curtaz et al., 2020; Garbuzova-Davis and Borlongan, 2021), it is suggested that stem cell therapy will ameliorate BBB damage via angiogenesis, reduce brain inflammation, and decrease brain tumor growth rate. Current research indicates that bone marrow-derived MSCs can ameliorate the effects of a compromised BBB (Menge et al., 2012; Silva et al., 2020; Tang et al., 2020), providing a protective effect by decreasing BBB permeability (Menge et al., 2012; Ge et al., 2019; Lin et al., 2019; Wang C. et al., 2020) and selectively localize to solid tumor foci in multiple organs after intravenous administration (Metz et al., 2013; Gutova et al., 2016; Yang M. et al., 2020). The compromised BBB permeability reduction is due in part to an increase in the collagen IV expression, providing the framework for endothelial-endothelial cell interaction, and an increase in occludin, promoting paracellular tight junction formation within the endothelial layer (Wang et al., 2015). TIMP1/CD63 axis promotes hNSC movement via the FAK/PI3K signaling and activation of integrin signaling via binding of CD63/integrin β1 complex, which in turn destabilizes the endothelial layer via inhibition of RhoA (Tang et al., 2020). MSCs produce TIMP3, a soluble factor which may inhibit VEGF-A, thus inhibiting further BBB breakdown (Menge et al., 2012). Furthermore, MSCs promote the up-regulation of Claudin-5, a tight junction protein essential to maintaining endothelial tight junctions, and increase MMP-9 levels resulting in restored BBB integrity in rat models (Lin et al., 2019). Stem cells combined with prodrug-activating enzymes can further induce the localization of a solid tumor formation, which may allow for tumor-localized chemotherapy (Metz et al., 2013). Furthermore, co-culture with hNSC decreases growth of brain metastasis from various cancers, decreases tumor volume and significantly prolongs survival (Seol et al., 2011; Hong et al., 2013; Mooney et al., 2021). BBB disruption caused by tumor progression exposes ECM substrates, which may cause hNSC attraction and localization, while anti-adhesive proteoglycans may protect undisturbed BBB (Mooney et al., 2021). NSC carrying therapeutic suicide genes, such as CE and CD, target tumor sites to activate chemotherapy drugs and induce cell death (Seol et al., 2011). Therapeutic use of allogenic human bone marrow-derived NSCs transfected with interferon-β (IFN-β) diminishes tumor growth, decreases metastasis, and increases longevity via AKT and ERK1/2 phosphorylation in the tumor cells (Bao et al., 2012; Hagenhoff et al., 2016; Romero-Trejo et al., 2021). Combination therapy of IFN-β/MSCs advantageously does not require immunosuppression and directly controls melanoma proliferation, allowing it to function as an anti-tumor therapy (Studeny et al., 2002; Du et al., 2019; Heo et al., 2019; Serakinci and Cagsin, 2019; Serhal et al., 2019). In the presence of tumors, MSCs possess the ability to proliferate due to the increase in paracrine growth factors that are present in the tumor microenvironment, but compete with local mesenchymal precursors (Studeny et al., 2002; Wang J. X. et al., 2020). IFN-β MSCs produce a direct anti-proliferative and metastatic effects on tumor cells by suppressing the cell proliferation gene expression and time-dependent mesenchymal–epithelial transition (MET) inhibition, respectively (Du et al., 2019; Heo et al., 2019). First, to demonstrate efficacy, direct implantation into an experimentally induced focal metastatic brain tumor may be required before evaluating an optimal route of SC transplantation. Afterward, using minimally invasive administration, such as intravenous (IV) or intra-arterial (IA), can portray the clinical expression identified by extensive infiltration on the majority of metastasized brain tumors. In terms of cell dosage, IA injections typically utilize lower doses of MSCs to effectively locate the metastatic cells. On the other hand, IV administration requires higher doses of MSCs to distribute over a wider area in a faster manner (Kabat et al., 2020; Rady et al., 2020). Additionally, the timing of implantation (e.g., acute vs. delayed metastasis) is critical to enhance clinical efficacy (Byun et al., 2020). When angiogenic genes are engineered into MSCs, especially if they have EPC phenotype, their transplantation serves as a viable treatment for attenuating brain inflammation and repairing the BBB (Antonucci et al., 2011; Miloradovic et al., 2020; Pawitan et al., 2020; Castelli et al., 2021; Li et al., 2021). For example, MSCs with overexpression of IL-10 shift macrophage expression from pro-inflammatory cytokines TNF-a and a reduction of activated macrophage and astrocytes correlated with a decrease in CD163 cells at the injury site in TBI models (Peruzzaro et al., 2019). MSC-EVs likewise reduced active astrocytes and microglial density in white matter following brain inflammation (Drommelschmidt et al., 2017). BMSCs with increased IL-12 influenced cell death by natural killer cell infiltration, arresting some cell growth in brain tumors (Hong et al., 2009). Moreover, establishing the therapeutic window for stem cell transplantation in brain metastasis should include evaluation of BBB repair and of decreased brain inflammation in both the acute and delayed injection time points of the mouse melanoma cell line B16F10. Along with the previously discussed endpoint assays (Zhang et al., 2018; Rady et al., 2020; Andrzejewska et al., 2021), and assessing the results of the grafted stem cells, we can outline the therapeutic potency of the stem cell transplants along with its safety in the brain. Potential advantages of cell therapy include the promotion of localized BBB angiogenesis and permeability, while reducing brain metastasis via anti-inflammatory processes. Additionally, optimizing timing and dose of stem cell transplant depending on their route of administration will allow safe and effective regimen in clinical trials for combatting melanoma derived brain metastatic brain cancer.

Due to the novelty of stem cell therapy to treat cancer, there are still many apprehensions of the effectiveness of this kind of treatment. Transplanted stem cells could possibly provide alternative angiogenic pathways for tumor growth and even become cancerous cells themselves (Razmkhah et al., 2019; Ahn, 2020; Atiya et al., 2020; Zhou et al., 2021). Thus, in order to advance stem cell-based treatments, rigorous examinations should delineate stem cell-induced BBB reconstruction from any adverse effects of inducing angiogenesis in cancerous brain cells, thereby localizing enhanced BBB permeability while mitigating tumor growth (Palmieri et al., 2007; Zhang et al., 2015; Le Rhun et al., 2019; Brighi et al., 2020; Hass et al., 2020; Lah et al., 2020; Yang M. et al., 2020).

## Caveats for Combining Stem Cell Therapy With Chemotherapy

Stem cell therapy has great success in the treatment of some cancers (Pascucci et al., 2014; Hu et al., 2018) and holds hope for other successful therapies as an adjunctive treatment (Chu et al., 2020). The effectiveness of cytotoxic therapy, such as in chemotherapy, may be restricted without the aid of other reagents (Lytle et al., 2021). The frequent use of HSC transplantation following high-dose chemotherapy (Copelan, 2006; Porfyriou et al., 2021) questions whether the treatment of stem cell therapy combined with chemotherapy may enhance therapeutics with heightened tumor targeting and elimination (Chu et al., 2020). However, when stem cell therapy is used in combination with chemotherapy for the treatment of cancer, this combination treatment can present contraindications and adverse events. Contradictory evidence exists on the long-term effectiveness of these combination therapies in some cancers as well (Table 1). These adverse events can present as stem cell contamination and toxicity, among others. Inflammation- and angiogenesis-relevant associated pathways can contribute to these challenges as well.

**TABLE 1:** List of studies comparing standard treatment or chemotherapy and combination therapy with stem cell therapy.

Study author and year published	Cancer type	Treatment	Significant findings
Stadtmauer et al. (2000)	Metastatic breast cancer	Maintenance verse high-dose chemotherapy and autologous SC transplantation	The study found no difference between the two groups’ outcomes.
Farquhar et al. (2005, 2016)	Breast cancer	Conventional verse high dose chemotherapy and BMSC/PSC autologous transplantation	The study found little to no difference between the two groups’ outcomes.
Namouni et al. (1997)	Retinoblastoma	Maintenance vs. high-dose chemotherapy and carboplatin, etoposide and cyclophosphamide and hematopoietic SC rescue	The study found improved outcomes in patients with interventional treatment at a 3-year survival disease-free time point. Patients that have co-occurring CNS disease did not have improved outcomes.
Motzer et al. (2007)	Metastatic germ cell tumors	Standard chemotherapy dose vs. high-dose chemotherapy and autologous hematopoietic SC	The study found no difference between the two groups’ outcomes.
Graham et al. (1997)	High-risk and recurrent pediatric brain tumors	Standard vs. high-dose chemotherapy and autologous marrow rescue with or without peripheral-blood SC	Interventional treatment shows it might improve outcomes.
Yalçin et al. (2015)	High−risk pediatric neuroblastoma	High−dose chemotherapy and autologous hematopoietic stem cell rescue	The study found no difference between the two groups outcomes.
Berry et al. (2011)	Breast cancer	High-dose chemotherapy and autologous hematopoietic stem cell transplantation	The study found improved outcomes in patients with high
Delaye et al. (2021)	Germ cell tumor with brain metastases	High-dose chemotherapy followed by infusions of autologous peripheral blood hematopoietic stem cells	The study found improved overall survival at 12 months.
Ferreri et al. (2017)	Primary CNS lymphoma	High-dose methotrexate-based chemotherapy followed by autologous peripheral blood stem-cell transplantation following	The study found the therapy effective at 2-year progression-free survival
Illerhaus et al. (2016)	Primary CNS lymphoma	High-dose methotrexate-based chemotherapy with autologous stem cell transplantation	The study found a complete response 30-days post treatment

### Evaluation of the Treatment of Breast Cancer With the Combination of Chemotherapy and Stem Cell Therapies

There is no conclusive data on high dose chemotherapy and stem cell long term benefits compared to standard therapy (Chen B. B. et al., 2016; Steenbruggen et al., 2020). While the combination of chemotherapy and stem cell therapy is hopeful as a treatment for cancer, there is conflicting literature at this time to unequivocally predict its efficacy in humans, as it can be dependent on type of chemotherapy, type of stem cells, and type of cancer (Somlo et al., 1994; VanderWalde et al., 2012; Qian et al., 2017). However, further insight into histopathologic and genetics may help evaluate how responsive a patient might be to these therapies (Zander et al., 2018). Reinfusion of peripheral blood progenitors is tolerated better, compared to bone marrow purging in patients with metastatic breast cancer and bone marrow infiltration (Myers et al., 1994). Risk factors that should be taken into account include patients that have received a doxorubicin treatment and breast cancer that has metastasized to the liver or lungs (Somlo et al., 1994). Bone marrow function returned to baseline in patients with high-risk breast cancer treated with autologous stem cell rescue following high dose CAW-16 polychemotherapy consisting of doxorubicin, etoposide, and cyclophosphamide (Somlo et al., 1994).

A common treatment related event is dose limiting toxicity, ranging from life threatening to death (Brockstein and Williams, 1996; Demirer et al., 2003). Treatment regimens complications that progressed from organ specific toxicity included renal failure and pneumonia syndrome (Berry et al., 2011). Treatment following high dose chemotherapy and autologous stem cell therapy may produce relapse (Somlo et al., 1994). There is no evidence of prolonged survival in patients with high-risk breast cancer who were treated with high dose chemotherapy in combination with autologous hematopoietic stem cell transplantation than a control group (Berry et al., 2011). High dose chemotherapy in combination with stem cells for breast cancer patients show long-term event-free survival, but an overall survival benefit is not significant (Berry et al., 2011). Likewise, high dose adjuvant chemotherapy with autologous hematopoietic stem cells has no significant overall survival value compared to control (Steenbruggen et al., 2020). However, there is evidence in breast cancers that involve at least ten axillary lymph nodes, HER2 negative breast cancer, and triple negative breast cancer (Steenbruggen et al., 2020).

There is no evidence that high-dose chemotherapy treatment in combination with stem cell therapy improves the median survival rate in comparison to the conventional-dose chemotherapy treatment in metastatic breast cancer (Stadtmauer et al., 2000). No significant difference accompanies the overall survival rate nor the time of progression between the participants in the treatment groups; however, further analysis revealed a potential survival advantage for women over 42 years old who only received conventional chemotherapy (Stadtmauer et al., 2000). Although not statistically significant, this finding warrants further investigation. In addition, the high-chemo stem cells group had a higher hazard ratio than the conventional-dose group. This indicates a lower likelihood of survival. Furthermore, the patient group who received a high-dose chemotherapy with stem cells showed a higher incidence of moderate and severe non-fatal adverse effects, suggesting that this type of treatment has a greater toxicity than the conventional chemotherapy approach (Stadtmauer et al., 2000).

Patients with intermediate to high grade lymphoma, who relapsed or were unresponsive to the conventional chemotherapy, demonstrated 40–50% long term disease-free survival if they receive a high-dose chemotherapy with autologous bone marrow transplant (ABMT) as the salvage treatment (Bosly et al., 1992; Douer et al., 1996). These findings offer evidence toward the efficacy of this treatment since patients who only receive conventional chemotherapy are typically considered to be incurable. In addition, patients with metastatic breast cancer who responded to the conventional-dose therapy and then underwent ABMT had a greater chance of achieving complete remission (Douer et al., 1996). Furthermore, newly diagnosed patients with stage II or III breast cancer and 10 or more lymph nodes had a lower 5-year recurrence rate if they were treated with ABMT compared to those treated with the standard therapy (Douer et al., 1996).

High-dose chemotherapy with autologous bone marrow stem cell transplantation can cause serious side effects (Farquhar et al., 2005). Six randomized trials tested the effectiveness of high-dose chemotherapy combined with autologous bone marrow stem cell transplantation to conventional chemotherapy in women with metastatic breast cancer (Farquhar et al., 2005). The high-dose treatment group survived significantly longer prior to experiencing a relapse of cancer. However, an increase in their overall survival rate is not seen when compared to the conventional treatment group. The side effects associated with treatment were worse in the high-dose chemotherapy with autograft treatment groups. The women that received high-dose chemotherapy with autograft were far more likely to experience severe adverse side effects such as hematological toxicity, sepsis, organ toxicity, and/or gastrointestinal toxicity (Farquhar et al., 2005). Even though in most cases these toxic effects were short-lived, the women reported experiencing continuous significant impairment 6–9 months after treatment (Farquhar et al., 2005).

### Evaluation of the Treatment of Brain Cancer With the Combination of Chemotherapy and Stem Cell Therapies

High-dose chemotherapy followed by infusions of autologous peripheral blood hematopoietic stem cells in patients with germ cell tumor with brain metastases found that the combination treatment had an overall survival of 12 months (Delaye et al., 2021). It should be noted that the results are limited as this sample size is small (Delaye et al., 2021). A phase 2 randomized trial, International Extranodal Lymphoma Study Group-32 (IELSG32), examined the efficacy of autologous peripheral blood stem-cell transplantation compared to whole-brain radiotherapy following high-dose methotrexate-based chemoimmunotherapy in patients with primary CNS lymphoma (Ferreri et al., 2017). This trial found that autologous stem-cell transplantation combination therapy was effective and there were no clinical significant differences to whole-brain radiotherapy in 2 year progression-free survival (Ferreri et al., 2017). Specifically, autologous stem-cell transplantation was associated with hematological toxicity and could be considered a possible option for patients following further research (Ferreri et al., 2017). Similarly, a phase 2 trial, Freiburg ZNS-NHL, examined the safety and efficacy of high-dose methotrexate-based chemotherapy with autologous stem cell transplantation with thiotepa and carmustine in patients with primary CNS lymphoma (Illerhaus et al., 2016). They found a majority of patients achieved a complete response in 30 days however toxicity was prevalent following induction of treatment (Illerhaus et al., 2016).

### Stem Cell Contamination

Peripheral blood stem cells (PBSCs) therapy may be beneficial to the cancer patients who are undergoing chemotherapy, in particular for those who receive the high-dose regimen due to its presumed role in the restoration of the immune system. However, there is a chance that during this procedure the PBSC cells become contaminated with the cancer cells, increasing the risk of micro-metastasis after the infusion of PBSC (Kasimir-Bauer et al., 2001). Patients with no or a low number of micro-metastatic cells in the bone marrow before and after chemotherapy, and no contamination of PBSCs, achieved complete remission (Kasimir-Bauer et al., 2001). This elucidates the potential implication of novel contaminated stem cell therapy to the overall outcomes to cancer treatment.

Tumor cells contained in autografts can contribute to relapse in certain types of cancer (Shimoni and Körbling, 2002). This highlights that although stem cells from blood may have less contamination from tumor cells compared to those of the bone marrow, they are still frequently contaminated. There is an association between the contamination of stem cell in autografts and a decrease in disease-free survival, which complements these results (Shimoni and Körbling, 2002).

**FIGURE 1:**
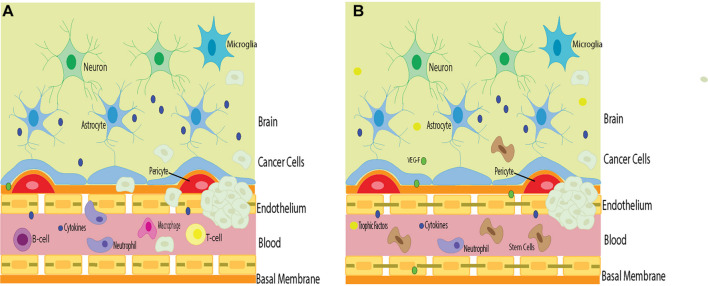
The barriers in stem cell therapy for brain cancers. Cancer cells in the brain circumvent the host immune surveillance, in part, due to their own tumor barrier. **(A)** Inflammation that accompanies cancer cell infiltration may compromise BBB permeability, allowing inflammatory cells to penetrate the brain and exacerbate tumorigenesis. **(B)** Stem cell therapy directed at repairing the BBB may prevent the influx of deleterious inflammatory cells into the brain and cancer cells and improve the surrounding environment. However, stem cells may also facilitate the potency of tumor barrier, allowing angiogenic and vasculogenic support to the cancer cells thereby aiding in tumorigenesis. Such risk-to-benefit outcome warrants a careful examination of advancing stem cell therapy for cancer treatment.

Stem cells can have a detrimental effect on the overall outcome of patients with certain types of cancer. Stem cell therapy combined with chemotherapy may exacerbate specific cancers. It is important to carefully consider such an approach. Adjusting the dosage, timing, and delivery of both strategies may be warranted to observe better outcomes. Identifying these patient populations who may be more vulnerable to worsened cancer outcomes also needs to be considered as part of differential diagnosis before contemplating such combined therapy for treating specific cancers. Because cancer cells possess their own tumor barrier, unique from the BBB, migration of stem cells and chemotherapy targeting the cancer cell tumor barrier and the BBB may circumvent triggering both barriers’ angiogenic and vasculogenic properties, which while beneficial for BBB, may aid the tumor barrier potentiation of cancer cells’ growth (Figure 1).

## Conclusion

Brain metastases may disrupt BBB and cause inflammation in the brain that exacerbates their growth rate. While it is shown that stimulating angiogenesis can lead to leaky blood vessels, we hypothesis in “3. Stem Cell Rescue of the Pathologic BBB” that metastatic cancer might have a similar effect of ameliorating similar to bone marrow-derived stem cells, expressing EPC phenotypes effect in stroke models, as we suggested in the following paragraph that stem cell therapy will ameliorate BBB damage via angiogenesis, reduce brain inflammation, and decrease brain tumor growth rate. Demonstrating this hypothesized stem cell treatment potentiates repair of the leaky BBB through stimulation of angiogenesis and vasculogenesis will be highly significant for basic science, translational, and clinical applications. Sequestration of inflammation within the brain via stem cell therapy may hinder the growth of malignant tumors and may present as a novel avenue for combating metastatic cancer and glioblastoma.

## Author Contributions

NS, AS, BB, MS, DC, CK, HZ, SA, Z-JW, FS, AL, GR, JV, AM, J-YL, and CB: conceptualization, literature analysis, writing- review, and editing. CB: supervision, project administration, and funding acquisition. All authors have read and agreed to the published version of the manuscript.

## Conflict of Interest

The authors declare that the research was conducted in the absence of any commercial or financial relationships that could be construed as a potential conflict of interest.

## Publisher’s Note

All claims expressed in this article are solely those of the authors and do not necessarily represent those of their affiliated organizations, or those of the publisher, the editors and the reviewers. Any product that may be evaluated in this article, or claim that may be made by its manufacturer, is not guaranteed or endorsed by the publisher.
